# Placental ischemia in pregnant rats impairs cerebral blood flow autoregulation and increases blood–brain barrier permeability

**DOI:** 10.14814/phy2.12134

**Published:** 2014-08-28

**Authors:** Junie P. Warrington, Fan Fan, Sydney R. Murphy, Richard J. Roman, Heather A. Drummond, Joey P. Granger, Michael J. Ryan

**Affiliations:** 1 Department of Physiology & Biophysics, University of Mississippi Medical Center, Jackson, Mississippi; 2 Department of Pharmacology and Toxicology, University of Mississippi Medical Center, Jackson, Mississippi

**Keywords:** AQP4, blood–brain barrier, CBF autoregulation, cerebrovascular abnormalities, edema, preeclampsia, pregnancy, tight junction proteins

## Abstract

Cerebrovascular events contribute to ~40% of preeclampsia/eclampsia‐related deaths, and neurological symptoms are common among preeclamptic patients. We previously reported that placental ischemia, induced by reducing utero‐placental perfusion pressure, leads to impaired myogenic reactivity and cerebral edema in the pregnant rat. Whether the impaired myogenic reactivity is associated with altered cerebral blood flow (CBF) autoregulation and the edema is due to altered blood–brain barrier (BBB) permeability remains unclear. Therefore, we tested the hypothesis that placental ischemia leads to impaired CBF autoregulation and a disruption of the BBB. CBF autoregulation, measured in vivo by laser Doppler flowmetry, was significantly impaired in placental ischemic rats. Brain water content was increased in the anterior cerebrum of placental ischemic rats and BBB permeability, assayed using the Evans blue extravasation method, was increased in the anterior cerebrum. The expression of the tight junction proteins: claudin‐1 was increased in the posterior cerebrum, while zonula occludens‐1, and occludin, were not significantly altered in either the anterior or posterior cerebrum. These results are consistent with the hypothesis that placental ischemia mediates anterior cerebral edema through impaired CBF autoregulation and associated increased transmission of pressure to small vessels that increases BBB permeability leading to cerebral edema.

## Abstract (precis)

Preeclampsia is associated with an increased risk for developing encephalopathies. A prevailing theory is that impaired cerebral blood flow autoregulation contributes to this process. Whether placental ischemia, commonly thought to be a major underlying factor in the development of preeclampsia, can cause impaired cerebral blood flow autoregulation is not clear. In this study, placental ischemia is experimentally induced to test this directly. The results show that placental ischemia in the pregnant rat causes marked impairment of cerebral blood flow autoregulation.

## Introduction

Preeclampsia is one of the leading causes of both maternal and perinatal mortality and morbidity, affecting 5–8% of all pregnancies in the United States. It is believed that the placenta plays a central role in the pathophysiology of preeclampsia since the symptoms remit after delivery of the placenta. While the placenta plays a critical role in the genesis of the disorder, multiple organs, including the brain, are commonly affected. Cerebrovascular events such as hemorrhagic stroke, cerebral embolism, and edema (Chakravarty and Chakrabarti 2002) contribute to about 40% of all preeclampsia/eclampsia‐related deaths (MacKay et al. 2001). Our laboratory recently reported that a rat model of placental ischemia, produced by reducing utero‐placental perfusion pressure (RUPP), exhibits cerebral edema (Ryan et al. 2011). However, the mechanisms by which placental ischemia produces cerebral edema are not known.

One potential explanation for cerebral edema following placental ischemia could be impaired autoregulation of cerebral blood flow (CBF) and increased transmission of the elevated systemic pressure to the small vessels and capillaries in the brain. The subsequent rise in capillary pressure is often associated with disruption of the BBB (Baumbach and Heistad 1985). We recently reported that placental ischemia leads to impaired cerebrovascular myogenic tone in isolated middle cerebral arteries in vitro (Ryan et al. 2011). However, it remains unclear whether a functional consequence of the reduced myogenic activity is impaired CBF autoregulation and blood–brain barrier (BBB) disruption.

In this study, we tested the hypothesis that placental ischemia leads to impaired CBF autoregulation, increased BBB permeability, regional cerebral edema and changes in the protein expression of tight junction and aquaporin channels.

## Materials and Methods

### Animals

Timed pregnant Sprague‐Dawley (CD) rats were obtained from Charles Rivers Laboratories and arrived at the Lab Animal Facilities at the University of Mississippi Medical Center on gestational day 11. All animal procedures were approved by the Institutional Animal Care and Use Committee at the University of Mississippi Medical Center. The rats were maintained on a 12‐hour light/12‐hour dark cycle and fed standard rodent chow and water ad libitum.

### Reduced uterine perfusion pressure model of placental ischemia

To induce placental ischemia, silver clips were surgically inserted around the abdominal aorta and each of the uterine artery branches from the ovarian arteries on day 14 of gestation as described previously (Granger et al. 2006). This method has been shown to reduce blood flow to the utero‐placental unit by ~40% in pregnant rats (Granger et al. 2006).

### Measurement of cerebral blood flow autoregulation

Normal pregnant and placental ischemic rats were anesthetized using 50 mg/kg Inactin (i.p) and 30 mg/kg ketamine (i.m) on gestational day 19. The trachea was cannulated using PE‐200 tubing and connected to a ventilator as described previously (Koehler et al. 2009; Renic et al. 2009). The right jugular vein was cannulated for intravenous infusion of saline to replace surgical fluid losses and graded infusion of phenylephrine. The heads of rats were then placed in a stereotaxic frame and secured in place using ear bars and a nose fastener. An incision was made on the top of the head to expose the skull. Using a drill, two closed, 4 mm × 4 mm, cranial windows were created approximately 3–4 mm lateral and 2 mm distal to Bregma by thinning the bone until the pial arteries were visible. A drop of paraffin oil was placed in each cranial window for optical coupling and blood flow was measured using Perimed dual channel laser Doppler flowmeter by placing the probes over the closed cranial windows. Mean arterial pressure (MAP) was continuously monitored via a carotid artery catheter. After surgery and a 30‐minute equilibration period, baseline readings were obtained (MAP, CBF, and exhaled CO_2_). Arterial Pco_2_ was maintained at 35 ± 2 mmHg for each experiment. MAP was elevated in steps of 20 mmHg from approximately 100–190 mmHg by graded intravenous infusion of phenylephrine. We focused on the higher range of blood pressures because preeclampsia is characterized by increased MAP and because the myogenic response is largely engaged at higher blood pressures. Once steady blood pressure was achieved, CBF measurements at the desired MAP were recorded. Blood pressure, CBF, and expired Pco_2_ were recorded continuously throughout the study.

### Determination of brain water content

On gestational day 19, rats were euthanized under isofluorane anesthesia and brains were quickly removed and separated into anterior and posterior cerebrum by cutting along the middle cerebral artery. Brain tissue anterior to the middle cerebral artery was designated as anterior cerebrum, while tissue posterior to the middle cerebral artery (without the cerebellum) was designated as posterior cerebrum. The tissue was weighed and then dried in an oven at 60°C for 72 hours, after which dry weight was obtained. Brain water content was then calculated as a percentage: ((wet weight − dry weight)/wet weight) × 100.

### Assessment of vascular permeability

A separate group of animals were utilized for determination of cerebral vascular permeability. Normal pregnant and placental ischemic rats were instrumented with jugular catheters on gestational day 18. Evans blue dye (1% solution in saline) was infused via jugular catheter so that each rat received 2 mg/kg of the dye which was allowed to circulate overnight. On gestational day 19, a sample of blood was obtained, and the brains were rapidly flushed with PBS at a rate of 15 mL per minute until the blood ran clear. The brains were then removed and divided into anterior and posterior cerebrum as described above, weighed, and snap frozen. Tissue and plasma samples were processed as previously described (Vital et al. 2010; Youssef et al. 2012). Briefly, 1 mL trichloroacetic acid (TCA, 50%) was added to samples, homogenized, and sonicated followed by centrifugation at 10,000 rpm for 30 minutes. Supernatants were then diluted 1:2 in 100% ethanol, vortexed, and loaded onto 96‐well plates. Plasma samples (10 *μ*L) were diluted in 50% TCA (990 *μ*L) and processed like the brain samples. Fluorescence was measured using a microplate reader at excitation 620 nm and emission 680 nm. Data are represented as concentration of Evans blue dye (ng/mL)/tissue weight (g)/plasma concentration (ng/mL).

### Assessment of plasma albumin concentration

Because of the high affinity of Evans blue for albumin, we measured plasma albumin to determine whether there were any differences in the amount of circulating albumin. A commercially available Rat Albumin ELISA kit was used and run following manufacturer's directions.

### Western blot analysis

The anterior and posterior brain regions were homogenized and equal concentrations of homogenate protein were loaded in 4–20% gradient gels, followed by electrophoresis and semi‐dry transfer. The membranes were blocked using Odyssey blocking buffer followed by overnight incubation in 1:2500 rabbit anti‐Claudin 1 abcam (Cambridge, MA), 1:1000 rabbit anti‐ZO‐1 Invitrogen (Grand Island, NY), 1:1000 rabbit anti‐Aquaporin 4 (abcam), or 1:250 rabbit anti‐Occludin (abcam) with either 1: 2000 mouse anti‐GAPDH (abcam) or 1:2000 mouse anti‐*β‐*actin antibodies. After three washes (5 minutes each), the membranes were incubated in donkey anti‐rabbit IRDye 700 and donkey anti‐mouse IRDye 800 for 45 minutes. The membranes were scanned using Odyssey scanner and the bands were analyzed using Image Studio Lite Version 3.1 Li‐Cor (Lincoln, NE). Relative expression was calculated as the ratio of fluorescence intensity of target protein to the intensity of GAPDH or *β*Actin and then further normalized to the corresponding expression in the normal pregnant rats.

### Statistical analysis

The significance of mean values in the normal pregnant and placental ischemic groups was determined using an unpaired t‐test. The CBF autoregulation data were plotted as the percent change in CBF versus MAP and the significance of differences in corresponding values measured at same level of MAP were determined using two‐way ANOVA with repeated measures followed by Bonferroni post hoc test. A *P* < 0.05 was considered statistically significant.

## Results

### Placental ischemia leads to impaired cerebral blood flow autoregulation

**Figure 1.**
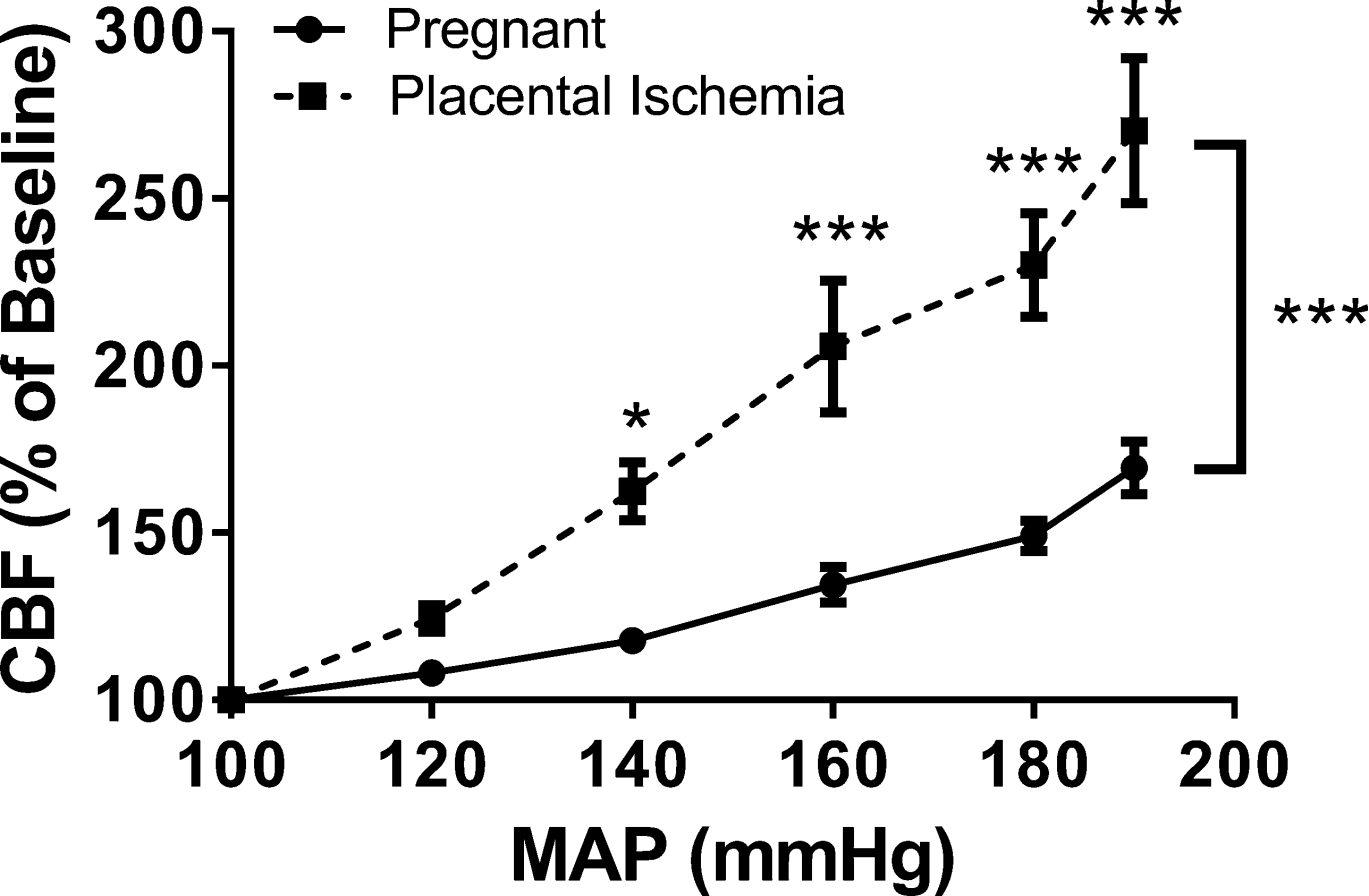
Cerebral blood flow autoregulation is impaired in placental ischemic rats. Cerebral blood flow, measured by laser Doppler flowmetry, increased significantly with blood pressure in placental ischemic rats compared to normal pregnant control rats. *P < 0.05, ***P < 0.001 versus normal pregnant group (*N* = 5 per group).

In order to assess CBF autoregulation, the relationship between the percent change in CBF versus MAP was plotted (Fig. 1). Two‐way repeated measures ANOVA showed that there was a significant interaction between group and MAP (*P* < 0.0001). There was a main effect of group (*P* < 0.001) and MAP (*P* < 0.0001) on CBF. Post‐hoc analysis revealed that CBF was significantly higher in the placental ischemic group at 140 (*P* < 0.05), 160, 180, and 190 mmHg (*P* < 0.0001) as compared to the levels seen in the normal pregnant group. Autoregulatory index was significantly different between the normal pregnant and placental ischemic group at all MAPs. At 190 mmHg, autoregulatory index was 0.77 ± 0.09 in the normal pregnant and 1.89 ± 0.24 in the placental ischemic group (*P* < 0.0001).

### Placental ischemia increases brain water content in the anterior cerebrum

**Figure 2.**
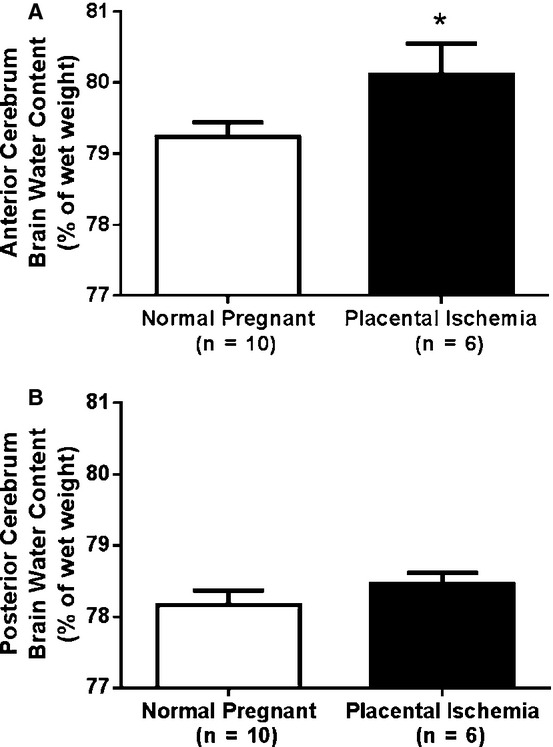
Placental ischemia induces cerebral edema. Brain water content was increased in (A) anterior cerebrum but not (B) posterior cerebrum of placental ischemic rats. Bars represent Mean ± SEM. *P < 0.05 versus normal pregnant group (*N* = 6–10 per group).

The water content in the anterior cerebrum was significantly higher in the placental ischemic group (80.11 ± 0.44%) compared to the normal pregnant rats (79.23 ± 0.21%) (Fig. 2**, ***P* <****0.05). In the posterior cerebrum, however, brain water content was similar (78.17 ± 0.21% in normal pregnant rats versus 78.46 ± 0.15% in placental ischemic rats; (*P* > 0.05). No difference in brain water content was observed in the cerebellum (data not shown).

### Placental ischemia increases cerebrovascular permeability in the anterior cerebrum

**Figure 3.**
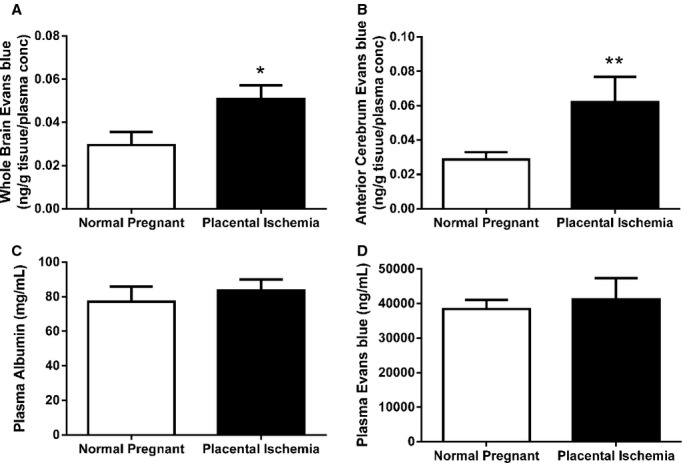
Blood–brain barrier permeability increases in placental ischemic rats. Evans blue extravasation into the brain tissue increased in (A) total brain and (B) anterior cerebrum of placental ischemic rats. (C) Plasma albumin concentration is similar between normal pregnant and placental ischemic rats. (D) Evans blue concentration in the circulation of pregnant and placental ischemic rats is not different. Bars represent Mean ± SEM (*N* = 4–9 per group).

To determine whether vascular leakage played a role in the edema formation, extravasation of Evans blue dye into the brain tissue was measured. Evans blue concentration in the whole brain was increased (*P* < 0.05) in the placental ischemic group (0.051 ± 0.006 ng/g tissue/concentration in plasma) compared to 0.030 ± 0.007 ng/g tissue/concentration in plasma compared to the normal pregnant rats (Fig. 3A). Evans blue concentration was increased (*P* = 0.01) specifically in the anterior cerebrum of the placental ischemic rats (0.062 ± 0.015 ng/g tissue/concentration in plasma; Fig. 3B) compared to normal pregnant rats (0.029 ± 0.004 ng/g tissue/concentration in plasma). However, there was no difference in permeability to Evans blue in the posterior cerebrum between normal pregnant and placental ischemic rats (data not shown). Plasma albumin was measured in order to determine whether the increased Evans blue extravasation resulted from differences in circulating albumin between the groups. No difference in plasma albumin (Fig. 3C) or plasma Evans blue concentration (Fig. 3D) was observed between the normal pregnant and placental ischemic rats (*P* > 0.05).

### Placental ischemia does not affect the expression of tight junction proteins

**Figure 4.**
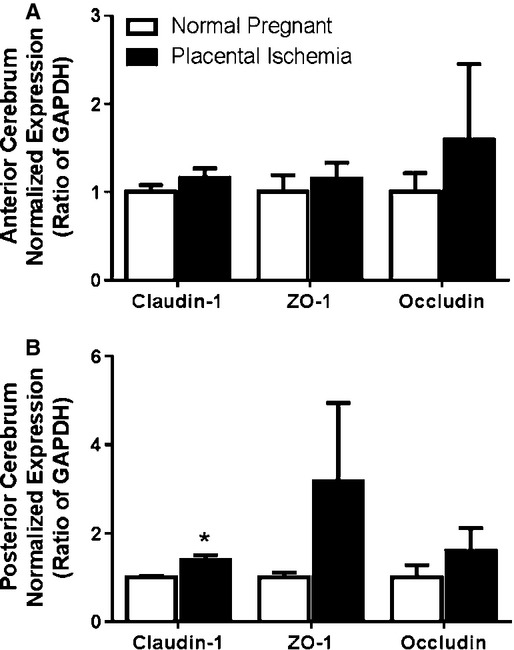
Expression of tight junction proteins in the (A) anterior cerebrum and (B) posterior cerebrum of placental ischemic rats. Quantitative analysis of Western blot for Claudin‐1, Occludin, and ZO‐1 normalized to beta‐actin or GAPDH. Blots are representative samples for respective groups – order of blots match the order of the bars. Bars represent Mean ± SEM. *P < 0.05 versus normal pregnant group (*N* = 6 per group).

To determine whether placental ischemia alters tight junction proteins as a contributing mechanism to the cerebral edema and vascular permeability, expression of claudin‐1, occludin, and zonular occludens‐1 was measured via Western blot. In response to placental ischemia, there was no significant difference in expression of claudin‐1, ZO‐1, or occludin in the anterior cerebrum (Fig. 4A). In the posterior cerebrum, claudin‐1 increased by 26% (*P* < 0.05), while ZO‐1 and occludin were unchanged in the placental ischemic rats (Fig. 4B).

### Aquaporin 4 protein expression is upregulated in the posterior cerebrum of placental ischemic rats

**Figure 5.**
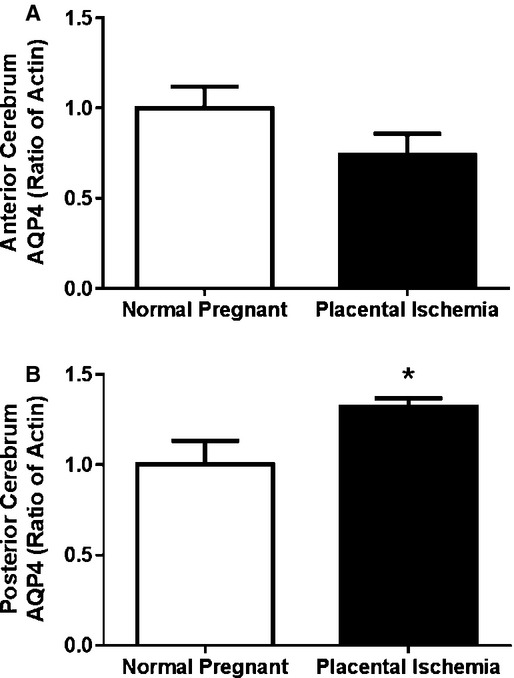
Aquaporin 4 protein expression is differentially altered in the anterior and posterior cerebrum of placental ischemic rats. Placental ischemia results in (A) no change in AQP4 expression in the anterior cerebrum and (B) increase in AQP4 expression in the posterior cerebrum. Blots are representative samples for respective groups – order of blots match the order of the bars. Bars represent Mean ± SEM. *P < 0.05 versus normal pregnant group (*N* = 6 per group).

The expression of AQP‐4 was assessed in the anterior and posterior cerebrum from placental ischemic and normal pregnant rats. There was no statistically significant difference in AQP4 expression in the anterior cerebrum (*P* > 0.05; Fig. 5A); however, AQP4 expression increased by 24% in the posterior cerebrum (*P* < 0.05; Fig. 5B) of placental ischemic rats compared to normal pregnant rats.

## Discussion

Cerebrovascular abnormalities are common to pregnancies that are complicated by preeclampsia, eclampsia, or the hemolysis, elevated liver enzymes, low platelet count (HELLP) syndrome (Zunker et al. 1995; Matsuda et al. 2005; Hirashima et al. 2006; Topuz et al. 2008; Aygün et al. 2010; Mitas and Rogulski 2012). Among the changes reported, blood–brain barrier disruption and cerebral edema are common findings; however, the mechanisms contributing to cerebrovascular changes during pregnancy are incompletely understood. The data in this study show that placental ischemia leads to impaired CBF autoregulation and increased BBB permeability, and thus provides important mechanistic insight for the cerebrovascular changes associated with preeclampsia. The increase in BBB permeability is not associated with changes in the expression of tight junction proteins; and AQP4, important for the resolution of edema, remain unchanged in the anterior cerebrum where significant edema was observed. These data are consistent with a mechanism whereby elevated MAP caused by placental ischemia impairs CBF autoregulation, resulting in increased transmission of pressure (hydrostatic) to the cerebral microvasculature leading to increased BBB permeability (Aygün et al. 2010; Cipolla et al. 2011).

We previously reported that placental ischemia in the rat impairs myogenic activity in isolated middle cerebral arteries (MCA) (Ryan et al. 2011). In that study, the diameter of MCA isolated from placental ischemic rats increased, almost passively, in response to increasing luminal pressure. However, whether the impaired myogenic activity in isolated vessels translates to impaired CBF autoregulation in vivo was not addressed. This study shows that the impaired myogenic activity functionally translates to the in vivo situation. Consistent with data from isolated MCAs (Renic et al. 2009), CBF increases passively in vivo in response to continually increasing blood pressure suggesting that placental ischemia leads to a marked impairment of cerebral blood flow autoregulation. The CBF data indicate that the autoregulatory index (% change in blood flow divided by the percentage change in perfusion pressure) is much greater than 1 in the placental ischemic rats as would be expected if the cerebral arteries simply failed to constrict. Our previous study showing that there is no difference in passive diameter or wall thickness between the normal pregnant and placental ischemic rats (Ryan et al. 2011) is consistent with the concept that pregnancy prevents or reverses hypertensive cerebral vascular remodeling (Cipolla et al. 2006, 2008). Therefore, the absence of hypertensive remodeling coupled with the markedly impaired cerebral blood flow autoregulation caused by placental ischemia may be an important mechanism for the increased susceptibility to encephalopathies in this patient population. This is a significant advance for the field because it directly links placental ischemia, a major factor in the pathogenesis of preeclampsia, to impaired CBF autoregulation. The previous work of others suggests that the forced dilation of cerebral vessels leading to increased CBF occurs at lower blood pressures during pregnancy (Cipolla and Osol 1998; Osol et al. 2002). However, those studies were conducted in normotensive late pregnant rats under conditions where blood pressure was raised acutely, and thus could not address the question of whether placental ischemia is a contributing factor.

A potentially important consequence of impaired CBF autoregulation is cerebral edema. The inability of the cerebral vessels to autoregulate in response to changes in arterial pressure can lead to increased arterial hydrostatic pressure favoring a net efflux of fluid from the arterial compartment, and potentially damaging the vasculature leading to increased BBB permeability. Indeed, our recent study showed that brain water content is increased in placental ischemic rats (Ryan et al. 2011). In this study, we extend these previous findings to show that the cerebral edema induced by placental ischemia is primarily characteristic of the anterior cerebrum. While numerous clinical studies report abnormalities in the parietal‐occipital lobe (Topuz et al. 2008; Aygün et al. 2010; Mitas and Rogulski 2012), there is evidence that the anterior/frontal lobe is affected as well (Riskin‐Mashiah and Belfort 2005). Additionally, in animal models of hypertension during pregnancy, cerebral edema has been reported in both the anterior and posterior cerebrum (Cipolla et al. 2012). Taken together, these studies demonstrate that cerebral edema during preeclampsia can affect both the anterior and posterior cerebrum.

Increased BBB permeability is a potential underlying mechanism that can result from impaired CBF autoregulation and lead to cerebral edema. Previous studies demonstrated that plasma from preeclamptic women is capable of inducing BBB disruption in isolated cerebral veins from nonpregnant rats (Amburgey et al. 2010; Schreurs and Cipolla 2013; Schreurs et al. 2013). Acute disruption of the BBB was also observed in patients with posterior reversible encephalopathy syndrome in response to abrupt increases in blood pressure (Aygün et al. 2010). Our results show that placental ischemia leads to increased BBB permeability in the anterior cerebrum in vivo, a finding that is in line with both clinical and basic research studies.

The BBB is formed from the association of several different cells such as endothelial cells, pericytes or smooth muscle cells, and astrocytic end feet to the cerebral vessels. The first interface between the blood and the brain tissue is formed from tight junctions between adjacent endothelial cells lining the lumen of the vessels. The tight junction consists of several proteins that maintain the integrity of the BBB such as claudins, occludins, and zonula occludens (ZO) (Reviewed in Liu et al. (2012)). Disruption of the BBB and reductions in expression of tight junction proteins lead to increased permeability and edema. Cerebral edema is characterized as either cytotoxic or vasogenic depending on whether the BBB remains intact (Betz et al. 1989). Cytotoxic edema occurs when water transport into cells through aquaporin (AQP) channels increases, leading to cell swelling, usually with the BBB still intact. Vasogenic edema involves physical disruption of the BBB resulting in increased water in the extracellular space. Reduction in the expression of tight junction proteins could contribute to increased BBB permeability. We therefore determined the relative expression of several important tight junction proteins in normal pregnant and placental ischemic rats. Our results suggest that increased tight junction protein expression in placental ischemic rats may protect the posterior cerebrum from edema. These data are consistent with findings that during late pregnancy, there is no difference in gene expression of claudin‐1, claudin‐5, occludin, and ZO‐1 in cerebral arteries (Cipolla et al. 2011).

Aquaporins (AQPs) are channel‐forming proteins through which water, glycerol, and other solutes enter cells (Verkman 2011). Three major AQPs are expressed in the brain: AQP1, AQP4, and AQP9, with AQP4 being the most abundantly expressed in the brain (Nicchia et al. 2004). AQP4 is mainly expressed in astrocytic end feet surrounding blood vessels, and ependymal cells that line the cerebral ventricles and is associated with edema resolution (Nicchia et al. 2004; Fukuda and Badaut 2012). In this study, we found that AQP4 protein is increased in the posterior cerebrum, which is protected from edema formation, but unchanged in the anterior cerebrum of placental ischemic rats. Compared to nonpregnant rats, late pregnant rats have increased AQP4 expression in the posterior brain but similar levels in the anterior brain (Wiegman et al. 2008), consistent with our findings in this study.

This study has important clinical implications given the evidence suggesting that CBF autoregulation in patients with preeclampsia and eclampsia is impaired, possibly accounting for the increased cerebral perfusion pressure in the anterior and posterior cerebral arteries in preeclamptic patients (Riskin‐Mashiah and Belfort 2005). For example, Zunker et al. reported increased CBF velocities in the middle cerebral arteries of patients with preeclampsia or eclampsia (Zunker et al. 1995). Moreover, patients who had preeclampsia in a previous pregnancy had poor dynamic CBF autoregulation (Janzarik et al. 2014). A small clinical study showed that 46% of preeclamptic and eclamptic patients studied exhibited cortical and/or subcortical lesions involving the occipital lobe; 72% of which had a watershed distribution that was hypothesized to result from acute fluctuations in blood pressure (Demirtaş et al. 2005). In addition, Manfredi et al. reported cortical and subcortical abnormalities in the posterior cerebrum of patients diagnosed with eclamptic encephalopathy as observed by CT and MRI techniques. The authors similarly hypothesized that this resulted from impaired CBF autoregulation that contributed to vasodilation, fluid extravasation, and hydrostatic edema (Manfredi et al. 1997). Therefore, the placental ischemic rat model utilized here will be an important tool to understand the mechanisms of impaired CBF autoregulation during preeclampsia.

## Significance and perspectives

Cerebrovascular complications of preeclampsia and eclampsia are commonly observed in the clinic and effective treatment and preventative measures remain elusive. In this manuscript, we showed that placental ischemic rats display impaired CBF autoregulation and that this is related to cerebral edema. The latter occurs primarily in the anterior cerebrum and is associated with increased BBB permeability potentially driven by a lack of edema resolution, evidenced by reduced AQP4 expression in the anterior cerebrum. These findings suggest that this rat model of placental ischemia is appropriate for assessing the mechanisms contributing to cerebral edema and impaired CBF autoregulation in the presence of placental ischemia. Future studies utilizing this model will focus on potential pathophysiological mechanisms that link placental ischemia with cerebrovascular dysfunction and brain edema.

## Acknowledgements

The authors would like to thank Kathy Cockrell, Marietta Arany, Grant Ross, and Edward Dent for their technical assistance.

## Conflict of Interest

None.
